# Identifying and Analyzing Topic Clusters in a Nutri-, Food-, and Diet-Proteomic Corpus Using Machine Reading

**DOI:** 10.3390/nu15020270

**Published:** 2023-01-05

**Authors:** Jacqueline Pontes Monteiro, Melissa J. Morine, Fabio V. Ued, Jim Kaput

**Affiliations:** 1 Department of Pediatrics, Ribeirão Preto Medical School, University of São Paulo, Bandeirantes Avenue, 3900, Ribeirão Preto 14049-900, Brazil; 2 Vydiant Inc., Dallas, TX 75201, USA

**Keywords:** machine reading, nutriproteomics, food proteomics, diet proteomics, nutrition proteomics, artificial intelligence, transformer-based language model

## Abstract

Nutrition affects the early stages of disease development, but the mechanisms remain poorly understood. High-throughput proteomic methods are being used to generate data and information on the effects of nutrients, foods, and diets on health and disease processes. In this report, a novel machine reading pipeline was used to identify all articles and abstracts on proteomics, diet, food, and nutrition in humans. The resulting proteomic corpus was further analyzed to produce seven clusters of “thematic” content defined as documents that have similar word content. Examples of publications from several of these clusters were then described in a similar way to a typical descriptive review.

## 1. Introduction

Nutrients and energy intake contribute to the initiation, progression, and outcome of multiple diseases [1]. However, many of the molecular mechanisms by which food components initiate diseases are not well defined, which hampers early detection of chronic diseases and the influence of nutrition on these processes. Transcriptomic [2], metabolomic [3], and proteomic technologies (this review) are increasingly used to probe subtle changes in cells and molecules in blood that are affected by different diets, nutrients, and food components.

Although blood is a key transport process to deliver metabolites to and from various organs, as well as the garbage system for removing unused end products and cell debris, both of which can be assessed from a venous blood draw. Sampling other human tissues, such as from adipose or muscle, is more invasive and has more challenges for sample preparation. Identifying biomarkers of exposure (e.g., [4,5]) is a primary goal of many of research studies and results of omic analyses of blood components can be used to develop predictive models that may explain the variability of nutrition response (e.g., [6])

Compared to genomic and transcriptomic technologies, high-throughput proteomic methods have been the most challenging to develop because of the broad range in concentration (particularly in the blood) and the extensive variation in physicochemical properties of proteins. Nevertheless, advances in proteomic technologies such as antibody-based multiplexed proximity extension assays [7], mass spectroscopic instrumentation and workflows [8,9], and DNA aptamer technologies [10] now permit the analysis of several thousands of proteins simultaneously. The Human Protein Atlas lists 4072 proteins in plasma detectable by mass spectroscopy (https://www.proteinatlas.org/humanproteome/blood+protein/proteins+detected+in+ms (accessed on 18 September 2022)) and Somalogic’s Somascan^TM^ platform can quantify over 7000 proteins (https://somalogic.com/specificity/ (accessed on 18 September 2022)).

The advancements in proteomic technologies are increasingly being used to identify clinically and nutritionally relevant biomarkers such as receptors, enzymes, and transporters. A PubMed search for (i) proteomics and nutrition, (ii) proteomics and diet, and (iii) proteomics and food listed 4970, 2766, and 10,485 citations, respectively (as of 18 September 2022). Classical manual methods of reviewing this literature would necessarily require restricting the search to more specific targets, eliminating the possibility of a comprehensive survey of proteomics in nutri-, food-, and diet-proteomics. An alternative approach is to use machine reading technology to extract and analyze the corpus of these topics.

We previously developed a natural language processing pipeline that parses, annotates, and analyzes ~37 M citations and publications in the National Library of Medicine (e.g., PubMed and PubMed Central) [11], as well as extracts semantically meaningful relationships between the labelled entities. The relation extractor module of the pipeline is built on a transformer-based language model [12] and fine-tuned to label the meaning and directionality of the extracted relationships. In this report, we used parts of the machine reading pipeline to identify, parse, and analyze all articles and abstracts on proteomics, diet, food, and nutrition in humans. The resulting proteomic corpus was analyzed to produce seven clusters of “thematic” content defined as documents that have similar word content. Examples of publications from several of these clusters are then described in a manner similar to a typical descriptive review. We propose that this machine-guided approach facilitates a more objective and systematic review of the articles in this domain.

## 2. Materials and Methods

### 2.1. Querying and Document Parsing 

The machine reading pipeline was essentially as described in [13] (excluding the relation extraction module) but briefly, the initial step in the pipeline development was to parse ~33 M citations in PubMed and ~2.4 M full-text records and annotate/index the concepts of interest (Table 1). The NCBI e-utils API service was used to fetch articles returned from the queries described in Table 1. The queries included both keywords and MeSH terms, so as to not rely exclusively on a set of query terms (as is the case with keyword search) but also to ensure inclusion of the latest articles (which are often missing from MeSH term search, due to the time lag in MeSH indexing). Further, within the MeSH term search, we queried using both the standard [MH] and [MAJR] tags, which restricts to articles with primary importance of the queried MeSH term. This allows separate assessment of the corpus sizes of these two searches. It is expected that the articles from the [MAJR] search are a strict subset of those from the [MH] search. The articles returned from the queries were parsed from XML to json using the PubMed parser Python library, and then the abstract and full text were split into sentences using the scispaCy en_core_sci_sm sentencizer model.

### 2.2. Document Annotation 

The proteomic-nutrition corpus was annotated using various approaches, depending on the entity type. The DNorm ([14] and https://www.ncbi.nlm.nih.gov/research/bionlp/Tools/dnorm/(accessed on 20 June 2020)) annotation tool from NCBI was used for disease annotations which provides integrated functionality for disease normalization to MeSH IDs. The GNormPlus ([15] and https://www.ncbi.nlm.nih.gov/research/bionlp/Tools/gnormplus/(accessed on 20 June 2020)) annotation tool from NCBI was used to integrate functionality for gene normalization to NCBI gene IDs. These results are discussed below and provided in an interactive Supplementary File.

### 2.3. Co-Mention Analysis 

An analysis was performed to identify sentences comentioning entity pairs of interest. Each comention relation is summarized in a network and/or table (examples below) with the following details: (i) the origin sentence and (ii) tagged entities (proteins or disease), labeled as V1 and V1 (vertex 1 and 2). Because comentions are nondirected, there is no semantic difference between V1 and V2. V1 and V2 are further described in terms of: (i) text_found which is the exact text representing the given entity, (ii) preflabel for the given entity (which serves to collapse synonyms for a common entity)—the preflabels (and not text_found) are the nodes in the network, and (iii) type entity which in this case are proteins or diseases. These results are discussed below with an interactive graph and a table of comention statements provided in the interactive Supplement Files SB.

### 2.4. Document Clustering 

To gain insight on the thematic content of proteomic-nutrition corpus identified by the pipeline, we performed document clustering using the term-frequency-inverse document frequency (tf-idf) metric. Briefly, this metric describes the importance of a given word in each (and every) document within the context of a larger corpus. tf-idf is highest when a word is common within a given document and rare in the rest of the corpus. Words with high tf-idf in document are therefore loosely analogous to keywords. We then used these numeric vectors to cluster the documents into thematic groups with K means clustering. For the K means clustering, we used a plot of the within-cluster sum of squares to decide on a suitable value for K. T-Distributed Stochastic Neighbor Embedding (t-SNE) was used for dimensionality reduction [16] and visualization [17]. Tf-idf vectorization, K means clustering, and t-SNE were all performed using the scikitlearn library for Python. An interactive version of the t-SNE plot and a summary table are provided in File S1, Figure S7.

### 2.5. Disease Annotation 

The Database for Annotation, Visualization, and Integrated Database (https://david.ncifcrf.gov/list.jsp (accessed on 20 June 2020)) was used for disease annotation.

## 3. Results

The information summarized in Section 3.1 through Section 3.5 is provided in more detail in supplement files SA and SB.

### 3.1. Query Results

**Figure 1:**
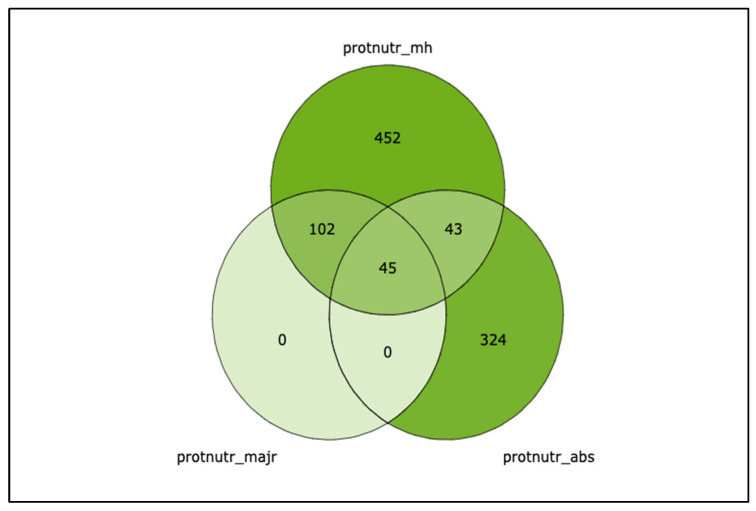
Venn diagram of unique abstracts with overlaps queries of nutrition proteomics. An interactive version of this figure with links to the PubMed abstracts is in the File SA.

The combined queries (Table 1) analyzed by the machine reading pipeline yielded 965 unique abstracts (Figure 1, interactive Figure in SA) and 273 unique full-text records (Figure 1). Only 39 of the 965 publications (4%) in this field are in journals with a SCImago Journal Ranking (SJR—https://www.scimagojr.com/aboutus.php (accessed on 30 September 2022) of four or above since 2002 (Figure S2 in File S1).

### 3.2. Document Annotation

**Figure 2:**
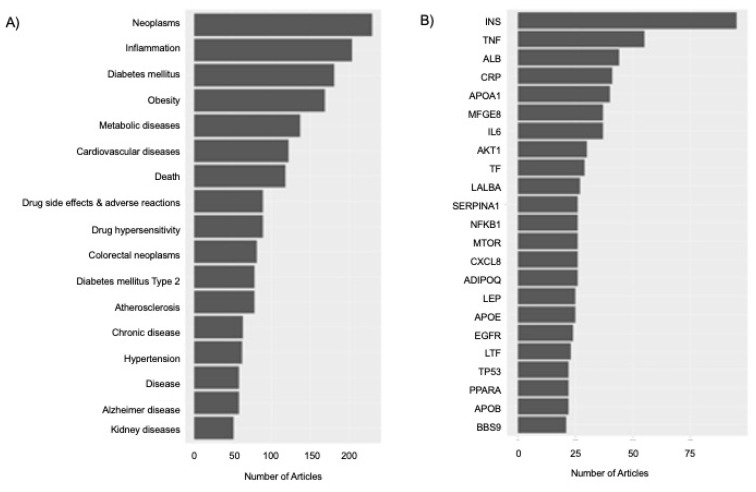
Document annotation of proteomic-nutrition corpus. (**A**) Diseases with 50 or more articles in the corpus. (**B**) Proteins with 20 or more articles. References for each disease or gene/protein can be obtained by clicking the bars in each interactive graph in supplementary File SA. The proteins are: ADIPOQ: adiponectin, C1Q and collagen domain containing; AKT1: AKT serine/threonine kinase 1; ALB: albumin; APOA1: apolipoprotein A1; APOB: apolipoprotein B; APOE: apolipoprotein E; BBS9: Bardet-Biedl syndrome 9; CRP: C-reactive protein; CXCL8: C-X-C motif chemokine ligand 8; EGFR: epidermal growth factor receptor; IL6: interleukin 6; INS: insulin; LALBA: lactalbumin alpha; LEP: leptin; LTF: lactotransferrin; MFGE8: milk fat globule EGF and factor V/VIII domain; MTOR: mechanistic target of rapamycin kinase; NFKB1: nuclear factor kappa B subunit 1; PPARA: peroxisome proliferator activated receptor alpha; SERPINA1: serpin family A member 1; TF: transferrin; TNF: tumor necrosis factor; TP53: tumor protein p53.

Annotation of the proteomic-nutrition corpus by DNorm [14] and GNormPlus [15] identified the diseases and gene/proteins, respectively, most often mentioned in the nutrition-proteomic corpus (Figure 2A,B, Supplement SA, Section 4). Importantly, these algorithms perform both tagging and normalization (i.e., collapsing of potentially long lists of tagged synonyms to a common label) of terms in the corpus. GNormPlus does not distinguish between genes and proteins in the document annotation.

### 3.3. Comention Analysis

**Figure 3:**
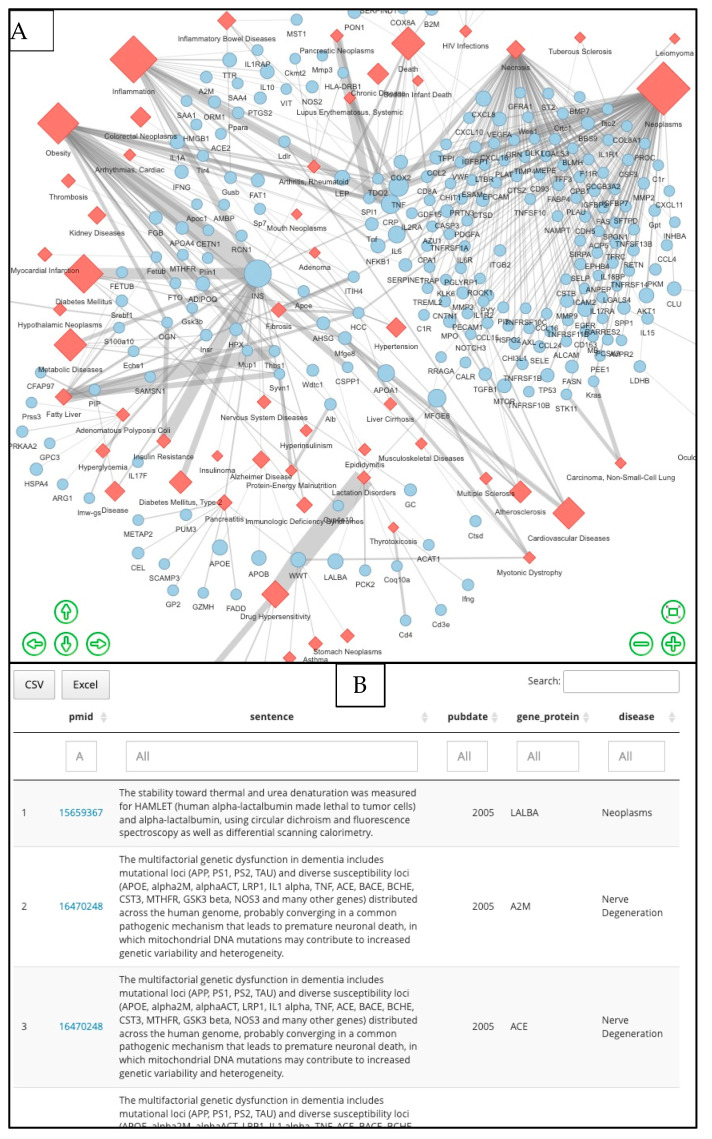
Protein–Disease Network and Protein–Disease Comention. (**A**) The protein–disease comention network from the nutrition–proteomic corpus, restricted to those protein–disease pairs with five or more supporting references (for plotting clarity). Blue circles are proteins and orange diamonds are diseases. The size of the symbols is proportional to the number of mentioning publications in the corpus. The edges are similarly scaled to represent the number of unique publications comentioning the two entities. An interactive network is in File SA, Figure S5.1. (**B**) Screenshot of File SA, interactive Table S5.2 with the PubMed ID (pmid), the extracted sentence, publication date, the gene/protein, and the disease. PMIDs retain links to the PubMed abstracts. Individual columns can be sorted (arrows in column headings) and searched.

Whereas Figure 2 provides an overview of the diseases and proteins studied in the nutrition-proteomic corpus, comention analysis identifies a link between two entities, in this case sentence-level comentions between proteins and diseases, which can be displayed as a network (Figure 3A) or in tabular form (Figure 3B). The nutrition-proteomic corpus consists of 5373 comentions between proteins and diseases (Table S5.2). Comentions have no direction or specific semantic meaning—the mentioned protein could affect the disease or conversely, the disease could over-express the protein—as two simple examples of many types of possible semantic relationships.

### 3.4. Document Clustering

**Figure 4:**
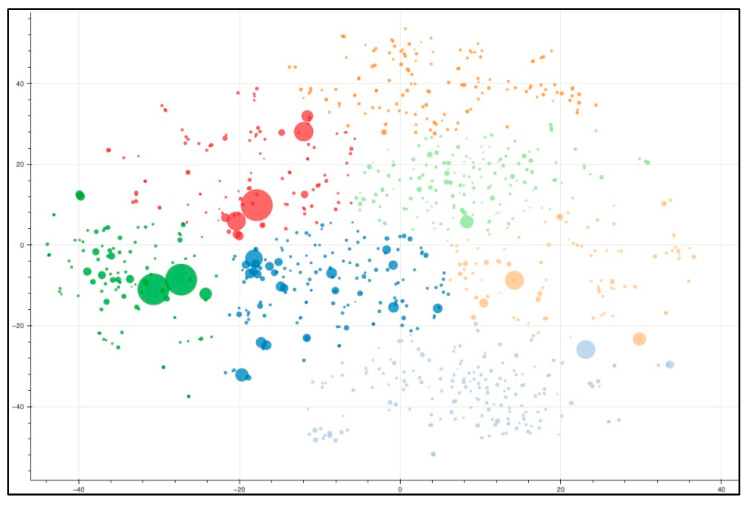
t-SNE cluster and visualization of nutrition-proteomic research themes. Each dot refers to a single article (click on dot in the interactive Supplement File SA, Figure S6.3 to open its PubMed abstract) and documents with similar word content are in close proximity. The size of the dot refers to the impact factor of the journal. The color of each dot refers to the cluster designation which in the text will labeled with a letter: (A) dark blue: protein, liver, diet, mouse, fatty, rat, disease, obesity, plasma, muscle; (B) light purple: milk, protein, peptide, human, infant, milkfat globular membrane (MFGM), colostrum, lactation, bovine, membrane; (C) orange: protein, plant, seed, food, allergen, gluten, wheat, soybean, crop, fruit; (D) light orange: protein, egg, salivary, plasma, child, biomarkers, saliva, disease, proteomics, proteome; (E) dark green: gut, probiotic, microbiota, protein, intestinal, cell, microbiome, bacterial, human, host; (F) light green: nutrition, nutritional, food, research, nutrigenomics, health, metabolomics, disease, genomics, science; (G) red: cell, protein, cancer, meat, colorectal, quality, proteomics, fish, study, muscle.

**Figure 5:**
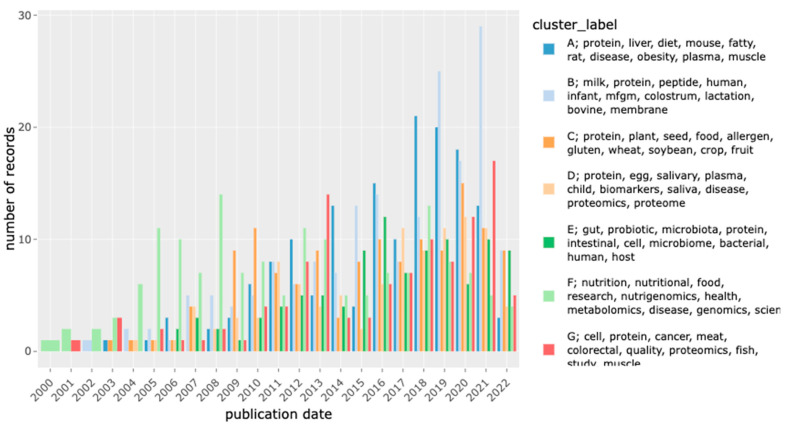
Cluster topics by year from 2000 to 2022. The interactive version of this figure is in Supplement SA, Figure S6.2.

An important step in many NLP analyses is the conversion of individual words and/or documents to numeric vectors—aka vectorization—which allows for mathematic analysis of the corpus by a variety of methods. For this analysis, we used the tf-idf metric as a document-level vectorization and used K-means analysis to identify seven clusters of these document vectors with increased similarity in keyword content within each cluster. Rather than using MeSH terms group or categorize the documents in our corpus, tf-idf was used because it is a purely data-driven and discovery-oriented approach that does not rely on a pre-defined set of categories that may or may not adequately describe the corpus. From the document clusters, we calculated the average tf-idf score per word within each cluster to identify the top 10 terms per group (Table 2). This “theme” variable describes the main topic of each cluster and provides a convenient filter for identifying publications of interest (Figure 4; the interactive version is in Supplement SA, Figure S6.3). The trends in the topic areas by year of publication is shown in Figure 5 (the interactive version is in SA, Figure 2).

### 3.5. Simple Quantitative Charactercistics of the Thematic Clusters

The publications in the clusters can be further analyzed for a variety of different “thematic characteristics.” For example, the occurrence and statistical overrepresentation of individual proteins (Table 3), disease associations (Table 4), or functional analysis using DAVID [18], STRING [19], or KEGG [20], or their functional analysis tools. The same proteins can be found in different clusters (see Supplement File SB for full list). The proteins in each cluster are (of course) related to and help drive the thematic content.

### 3.6. Disease Annotation

The DAVID Functional Annotation Tool [18] was used to analyze disease associations of the proteins in each of the thematic clusters to provide more context on the key word results used to define the clusters (Table 4). In general, the diseases found were consistent with the keywords identified by top tf-idf keywords in each cluster. As expected, the larger the cluster, the more diseases are associated with the proteins. Cluster C is an exception because many of the publications in this theme are related to milk production in agricultural animals and humans.

### 3.7. From Group Level Data to Individual Papers

Analyses of all articles in the nutrition-proteomic corpus (Figure 1, Figure 2, Figure 3 and Figure 4 and Table 1 and Table 2) or characterizations of the publications grouped by the tf-idf cluster analysis (Table 3 and Table 4) provide a metaphorical ~30,000 foot or ~1000 ft view of the extracted articles. Individual abstracts or publications can also be viewed and further analyzed by manual text mining typical for preparing publications, systematic reviews, and meta-analysis. For illustrative purposes, Table 5 compares the top words of the thematic cluster with the top words in the document for all the publications described in the following section. By definition, the top cluster words will also show more frequent use within most or all documents in the cluster. However, it is not necessarily the case that top cluster words should also be top document words for each document in the cluster. Selected articles from each cluster are discussed to show the utility of the pipeline and subsequent data-driven cluster analysis. We did not focus on a particular topic for each cluster, but selected articles based current interest to clinical and research nutritionists interested in proteomic analysis of health, disease, and treatments. 

#### 3.7.1. Cluster A—Protein, Liver, Diet, Mouse, Fatty, Rat, Disease, Obesity, Plasma, Muscle

Nutritional research is increasingly focused on measuring the effect dietary patterns on health and disease processes rather than how specific foods or isolated nutrients alter physiology. 

Proteomic profiles in 1713 participants of the Framingham Heart Study were analyzed in three different dietary patterns, (i) Alternative Healthy Eating Index (AHEI), (ii) the Dietary Approaches to Stop Hypertension (DASH) diet and (iii) the Mediterranean-style (MDS) [21]. DNA-based aptamers (SOMAscan) were used to find unique associations between dietary patterns and 17 plasma proteins with AHEI, 52 with DASH, and 3 with MDS. Significant proteins enriched biological pathways involved in cellular metabolism/proliferation and immune response/inflammation, providing insights into the molecular mechanisms mediating diet-related disease. Although the specific proteins may become biomarkers of these three dietary patterns, the results need replication in independent populations.

A separate study examined the AHEI and modified versions of the Mediterranean-style Diet Score (mMDS) and mDASH in 6360 participants (mean age 50 years; 54% women) in the same Framingham Heart Study [22]. The proteomic analysis used a modified sandwich ELISA method multiplexed on a Luminex xMAP (Sigma-Aldrich, St. Louis, MO, USA). The associations between diet and 71 candidate cardiovascular disease (CVD)-related proteins were examined in individuals against the three diet quality scores. Mediation analysis identified proteins that mediated the associations between diet and incident CVD and all-cause mortality. The results indicated that a healthy diet is associated with circulating cardiovascular disease-related protein biomarkers, largely representing regulators of inflammatory pathways in the group of middle-aged and older participants. Four proteins—B2M (beta-2-microglobulin), GDF15 (growth differentiation factor 15), sICAM1 (soluble intercellular adhesion molecule 1), and UCMGP (uncarboxylated matrix Gla-protein)—may mediate the association of diet with health outcomes.

The CORonary Diet Intervention with olive oil and cardiovascular PREVention [CORDIOPREV] study [24] evaluated the effect of two healthy dietary models (a Mediterranean diet and a low-fat diet) on endothelial function, measured by flow-mediated dilation (FMD), in patients with coronary heart disease (CHD). Patients with CHD following the Mediterranean diet had higher FMD compared with those on a low-fat diet, regardless of the severity of endothelial dysfunction. The Mediterranean diet also led to better endothelial function, enhanced endothelial repair mechanisms, and a reduction in the mechanisms associated with endothelial damage. Patients who consumed the Mediterranean diet had lower miR181c-5p levels as compared with a low-fat diet, inhibiting the proapoptotic action of this miRNA. miR181c-5p is also implicated in ROS synthesis, so the reduction in the levels of this miRNA would lead to a decrease in GPx3, an enzyme that catalyzes the reduction in ROS, as was observed in another proteomic study [36]. The Mediterranean diet may better modulate endothelial function compared with a low-fat diet and is associated with a better balance of vascular homeostasis in CHD patients, even in those with severe endothelial dysfunction.

Proteins from liver tissue samples from controls and patients with parenteral nutrition (PN)-associated liver disease (PNALD) were analyzed with the Isobaric Tag for Relative and Absolute Quantitation (iTRAQ)-based quantitative proteomics method [23]. A total of 112 proteins were found to be differentially expressed, of which 73 were downregulated, and 39 were upregulated in tissues from the PNALD group. These proteins were associated with mitochondrial oxidative phosphorylation, hepatic glycolipid metabolism (primarily involved in glycogen formation and gluconeogenesis), and oxidative stress involved in antioxidant change, such as, CYP2B6, DDAH1, and NDUFA1 that were significantly downregulated, and FABP5 and CAPG that were upregulated in the PNALD group, compared to the control group. This study identified candidate proteins as future PNALD biomarkers or therapeutic targets related to long parenteral nutrition therapy.

A follow-on study of the DioGENES weight maintenance study [37] analyzed 173 glycemic responders versus 201 glycemic nonresponders who had previously lost >8% of their body weight on a 800 kcal/d for 8 week diet. The two groups were comparable at baseline for body composition, glycemic control, adipose tissue transcriptomics, and plasma ketone bodies, but they differed significantly in their response to LCD, including improvements in visceral fat, overall insulin resistance (IR), and tissue-specific IR. Proteomics analysis was conducted using DNA aptamers (version 1 of Somascan, SomaLogic, Boulder, CO, USA) which revealed that total ApoE, ApoE2, ApoE3, and ApoE4 differed significantly between responders and nonresponders. In addition, proteomic pathway analyses highlighted other proteins involved in lipoprotein metabolism (APOA1, BMP1, FABP3 and ANGPTL4) that are key markers in obesity and NAFLD (e.g., [38,39]). Given the link between NAFLD, insulin resistance, and lipid metabolism, these markers, as well as other adipo- and hepatokines deserve further investigation in the context of weight loss and glycemic improvements following LCD intervention.

#### 3.7.2. Cluster B—Milk, Protein, Peptide, Human, Infant, Milk Fat Globule Membrane, Colostrum, Lactation, Bovine, Membrane

Milk fat globules (MFGs) are a mixture of proteins and lipids with nutraceutical properties related to the milk fat globule membrane (MFGM). Given the similarities between the human and bovine MFGM and the bioactive properties of MFGM components (rev. in [26]), several attempts have been made to supplement infant formulas (IFs) with polar lipid and protein fractions of bovine. The most shared proteins across species were involved in protein/vesicle-mediated transport, along with major MFGM proteins such as BTN, ADPH, FABP, and MUC1. The main difference regarding human MFGM proteome was a higher enrichment in enzymes involved in lipid catabolism [27] and in a set of immune response proteins [28]. The similarities between human and cow MFGM proteome and molecular functions, suggesting that bovine milk, and more specifically bovine MFGM proteins, could be used as a supplement in infant formulas (rev in [26]). 

Phosphorylation is a widespread posttranslational protein modification involved in regulation of many biological processes. Liquid chromatography mass spectroscopy (LC-MS/MS) quantitatively analyzed phosphorylation sites in human milk and colostrum fat globule membrane (MFGM) proteins [29]. A total of 71 phosphorylation sites in 48 human MFGM proteins differed between stages. These 48 phosphoproteins were mainly associated with immune-related processes. The majority of these phosphoproteins were involved in 16 of KEGG pathways including insulin, AMPK, Ras, PI3K-Akt, ErbB, apelin, and chemokine signaling pathways. SPRING functional protein association network analysis suggested more immune system process-related phosphoproteins in human colostrum MFGM than in mature MFGM, probably because of the important role that colostrum has in building the immune system of newborns [29]. 

Different storage conditions, and particularly temperature variation may affect the stability of the human milk proteome. More specifically, 22 of 110 quantifiable proteins significantly decreased after 48 h at 4.9 and 6.0 °C (rev. in [30]). Pasteurization of human milk changes milk components such as immunoglobulins and lactoferrin (rev. in [30]). An additional variable in milk composition may be caused by environmental factors. For example, children who grow up on farms are at lower risk of developing childhood atopic disease, which is called the “farm effect” [40]. A quantitative glycoproteomics analysis of 54 milk samples from Rochester urban/suburban and Old Order Mennonite mothers identified differences in 79 *N*-glycopeptides from 15 different proteins, including many involved in immune function [31]. These findings highlight the importance of understanding and recording metadata such as storage conditions, processing, handling, and origin of samples not only for proteome research, but also for nutritional content of milk products. 

#### 3.7.3. Cluster C—Protein, Plant, Seed, Food, Allergen, Gluten, Wheat, Soybean, Crop, Fruit

The consumption of bread wheat (Triticum aestivum ssp. aestivum) products can cause celiac disease (CD), allergic reactions, and nonceliac wheat sensitivity (NCWS) (rev. in [32]). The proteomes of 15 representative varieties of the bread wheat flour and a spelt subspecies spelt were analyzed using nano LC–ESI–MS/MS. Although 81 proteins (e.g., alpha-amylase inhibitors, serpins, gliadins, glutenins, and Bowman–Birk trypsin inhibitor) had high heritability, protein expression was largely driven by environmental effects. Of the 3050 proteins expressed in spelt, 1555 proteins were differentially expressed depending on field locations. Similarly, 1166 of 2770 proteins in bread wheat showed differential expression, underlining the large environmental impact caused by type of nitrogen fertilization, weather conditions, and soil types (rev. in [32]). 

Traditional food plants (TFPs) typically consumed by rural indigenous communities across the globe have been gaining increasing importance in maintaining genetic diversity as climate changes alter local environments (rev. in [33]). Proteomic analysis of samples of *Manihot esculenta* (*cassava*) obtained during cold or drought stress identified changes in levels of protein such as ATP synthase subunit beta, rubisco activase (RCA), rubisco, phosphoglycerate, chaperone peroxiredoxin, heat shock protein, glutathione transferase, and the drought-induced Di19-like protein (rev. in [33]). In addition to improving sustainable growth characteristics, proteomic analysis can also identify processes for improving the nutritional content of plants. For example, the expression of the phytoene synthase (EutPSY) gene was found to be correlated with the higher accumulation of lycopene in the silverberry (E. *umbellate*) (rev. in [33]). Understanding the proteomic changes caused by climate stress or the pathways responsible for levels of nutritionally important metabolites may allow for rapidly improving resilience and nutritional content in these and other plants using modern genetic modification procedures (e.g., CRISPR). 

#### 3.7.4. Cluster E—Gut, Probiotic, Microbiota, Protein, Intestinal, Cell, Microbiome, Bacterial, Human, Host

No accepted diagnostic markers are known for the three subtypes (i.e., diarrheal, constipation, or mixed) of inflammatory bowel syndrome (IBS). Tandem mass tag (TMT)-based proteomics of samples were obtained from intestinal mucosa samples during colonoscopies of IBS-diarrheal and controls [34]. Eighty differentially expressed proteins were with relative expression levels of 48 up-regulated at 1.2 or greater (*p* < 0.05) and 32 down-regulated at 1.2 or greater (*p* < 0.05). The identified proteins were significantly enriched in the nutrient ingestion pathways related to immune molecules and to FODMAP (fermentable oligosaccharides, disaccharides, monosaccharides, and polyols) metabolism including methanethiol oxidase (SELENBP), V-Set, and immunoglobulin domain containing 2 (VISG2), 7-dehydrocholesterol reductase (DHCR7), B cell receptor associated protein 31 (BCAP31), peroxisome proliferator activated receptor alpha (PPARA) and delta (PPARD), galactose mutarotase (GALM), carbonic anhydrase 2 (CA2), immunoglobulin kappa variable 1–33 (IGKV1-33), and fatty acid binding protein (FABP1), among others. These results confirmed changes in immune function pathways but also identified potential new explanations for why low FODMAP diets are not tolerated by IBS-D patients. 

#### 3.7.5. Cluster G—Cell, Protein, Cancer, Meat, Colorectal, Quality, Proteomics, Fish, Study, Muscle

Intermittent fasting has been linked to improved health outcomes, but the molecular mechanisms are unknown. A mass spectroscopic proteomic analysis was conducted of serum samples from 14 healthy subjects who fasted from dawn to sunset (i.e., greater than 14 h daily) for 30 consecutive days but otherwise were not calorically restricted [35]. The untargeted serum proteomic profiling identified upregulation of key regulatory proteins involved in glucose and lipid metabolism, circadian clock, DNA repair, cytoskeleton remodeling, immune system, and cognitive function. The identified proteins are associated with protective effects for cancer, metabolic syndrome, inflammation, Alzheimer’s disease, and several neuropsychiatric disorders. For example, a 9-fold increase was found for the large tumor suppressor kinase 1 (LATS1) and an 11-fold increase in NR1D1 nuclear receptor subfamily 1 group D member 1 (NR1D1), with a significant reduction in the amyloid beta precursor protein (APP), beta-1,4-galactosyltransferase 1 (B4GALT1), and ArfGAP with SH3 domain, ankyrin repeat, and PH domain 1 (ASAP1) at the end compared to before the 30-day intermittent fasting period.

## 4. Discussions and Conclusions

### 4.1. Data-Driven Analysis and Organization of Literature

NLP methods have been rapidly adopted in the biomedical literature and, increasingly, the Electronic Health Record space (e.g., [41]), particularly since the development of the widely impactful transformer-based deep learning models [42]. A selection of applications of NLP for information extraction in biomedical/clinical text include: tagging [43], normalization and linking of biomedical entities [14], relation extraction [44], and event extraction [45]. To our knowledge, NLP has not been used for preparing narrative or systematic reviews although several reports compared ML approaches to guide initial selection of the literature corpus rather than using ML for downstream information extraction from the retrieved documents [46,47,48]. A PubMed knowledge graph connected (1) authors, their educational background, funding data, and affiliation history with (2) the diseases, drugs, genes, species, and mutations identified in their corpus of abstracts [49]. An alternative approach to literature retrieval, which we have applied in the domain of whole-food and nutrient effects on human disease [11], is to individually index all foods (to the extent reasonably possible) and nutrients in all biomedical abstracts and full text, and retrieve all articles referencing any food or nutrient. 

Identification of the (in this case) nutrition-proteomic corpus presented here is naturally specific to the exact search terms described in Table 1 and, therefore, some proteomic studies in PubMed/PMC will not be the identified by the pipeline if they do not match the specific search terms (in this case, Table 1). As examples, our team previously used samples from a micronutrient intervention in healthy children aged from 9 to 13 years old [6] for three DNA-aptamer-based serum proteomic studies. One study explored the relationship of 117 pro-inflammatory proteins with different levels of DNA damage and EPA and DHA levels [50]. A second study explained levels of serum LTAH4 with plasma levels of cobalamin, riboflavin, pyridoxal, and homocysteine [51]. The third study identified 20 differentially expressed proteins (with iTRAQ technology) between 10 individuals with lower triglycerides, LDL, and VLDL compared to 10 individuals with higher lipid profiles [52]. Although these reports described analysis of nutritional biomarkers in serum (lipid profile, vitamin levels, and hundreds of other metabolites [6]), none of these papers appeared in the nutrition-proteomic corpus because they did not include the terms “nutrition” or “nutritional” (or corresponding MESH term) in the abstract and publication. Hence, they did not match our queries. Different search terms such as nutritional or serum biomarkers, blood metabolite levels, or lipid profiles and proteomics would identify those documents focused on those topics. However, for this review, we aimed to maintain a focus on articles with explicit reference to the field of proteomics in nutrition, diet, and food research. Our analyses did not assess the populations (e.g., description of participants in study), intervention (e.g., diets, drugs, or other), comparator (e.g., diseased versus control, or high fat group versus low fat group), or outcome (e.g., decreased biomarker or disease symptom)—PICO [53]—for each study in the corpus, which was beyond the scope of the topic for this review.

We used K-means cluster analysis to identify seven “themes” in the extracted nutri-proteomic corpus. Others have reported methodological studies that compared various analytical methods (i.e., tf-idf, latent semantic analysis, topic modeling, self-organizing maps, and poison-based [46] or Gaussian mixture model [54]) to identify thematic cluster documents extracted using MESH terms [46] or the BioBERT language model [54]. Regardless of the methodological approach, cluster analysis further contextualizes documents identified by machine reading technologies.

### 4.2. Conclusions

The use of high-throughput quantification and analysis of proteins in response to nutrition, food, or diet differences has been increasing rapidly with the development of improved mass spectroscopic sample workflows (e.g., [8]), molecularly tagged antibody technology (OLink [7]), and aptamer technologies [10]. The machine learning pipeline used here identified 945 publications on proteomics in the domain of nutrition, food, and diet. Reviewing this number of publications requires some ideally unbiased type of selection method and criteria. Unlike manual sorting of this corpus, publications were grouped into thematic clusters based on their word usage (i.e., tf-idf). Each cluster of publications could be the basis of a narrative, systematic, or meta-analytic review. 

## Figures and Tables

**Table 1:** Machine Reading Search Terms.

Query	PubMed Terms ^1^
protnutr_mh	Proteomics [MH] AND “Diet, Food, and Nutrition” [MH] and Human [MH]
protnutr_majr	Proteomics [MAJR] AND “Diet, Food, and Nutrition” [MAJR] and Human [MH]
protnutr_ab	(proteomics [TIAB] OR “DNA aptamer” [TIAB] OR Somascan [TIAB]) and (“Nutrition” [TIAB] OR “Nutritional” [TIAB]) AND (Human [MH] OR Human [TIAB] or individuals [TIAB] or patients [TIAB] or participants [TIAB] or subjects [TIAB])
**Query**	**PubMed Central Terms**
protnutr_mh	Proteomics [MH] AND “Diet, Food, and Nutrition” [MH] AND Human [MH] AND (open access [filter] OR author manuscript [filter])
protnutr_majr	Proteomics [MH] AND “Diet, Food, and Nutrition” [MH] AND Human [MH] AND (open access [filter] OR author manuscript [filter])
protnutr_ab	(proteomics [Abstract] OR “DNA aptamer” [Abstract] OR Somascan [Abstract]) and (“Nutrition” [Abstract] OR “Nutritional” [Abstract]) AND (Human [MH] OR Human [TIAB] or individuals [Abstract] or patients [Abstract] or participants [Abstract] or subjects [Abstract]) AND (open access [filter] OR author manuscript [filter])

^1^ [MH] refers to regular MeSH tag, [MAJR] refers to MeSH tag of primary importance to the paper (as designated by National Library of Medicine analyst), [TIAB] refers to ‘title/abstract’. The [TIAB] query captures, and in particular, articles that do not have MeSH tags. Subquery (open access [filter] OR author manuscript [filter]) is added to each query, to limit to those articles freely available to commercial entities.

**Table 2:** Themes of Nutrition Proteomic Research ^1.^

Cluster	Theme	Cluster Size	Median SJR	Median Year	Earliest	Latest
A	protein, liver, diet, mouse, fatty, rat, disease, obesity, plasma, muscle	112	1.189	2017	2001	2022
B	milk, protein, peptide, human, infant, milk fat globule membrane, colostrum, lactation, bovine, membrane	101	1.189	2017	2006	2022
C	protein, plant, seed, food, allergen, gluten, wheat, soybean, crop, fruit	181	1.279	2018	2002	2022
D	gut, probiotic, microbiota, protein, intestinal, cell, microbiome, bacterial, human, host	158	0.934	2012	2000	2022
E	nutrition, nutritional, food, research, nutrigenomics, health, metabolomics, disease, genomics, science	104	1.085	2017	2004	2022
F	cell, protein, cancer, meat, colorectal, quality, proteomics, fish, study, muscle	156	1.323	2017	2003	2022
G	protein, egg, salivary, plasma, child, biomarkers, saliva, disease, proteomics, proteome	133	1.189	2016	2003	2022

^1^ t-SNE derived clusters (A-G); theme is pipeline-derived keywords. Cluster size is number of proteins in cluster. SJR is Scimago Journal Ranking. Median year of publication of all papers in the cluster with earliest and latest publication in the cluster (as of August 2022). Visualization is in Figure 4 and the interactive version in Supplement SA, Figure S2.

**Table 3:** Protein Enrichment Per Cluster ^1^.

Cluster	Protein_ID	Name	#/Cluster	*p*-Value
A	INS	insulin	21	3.14 × 10^−7^
A	ADIPOQ	adiponectin, C1Q and collagen domain containing	5	7.43 × 10^−3^
A	APOE	apolipoprotein E	5	1.84 × 10^−2^
A	HP	haptoglobin	4	2.75 × 10^−2^
A	Insr	insulin receptor	3	4.03 × 10^−2^
A	PRKAA2	protein kinase AMP-activated catalytic subunit a2	3	4.03 × 10^−2^
A	Srebf1	sterol regulatory element bindingTF1transcription	3	4.03 × 10^−2^
A	TNF	tumor necrosis factor	5	4.88 × 10^−2^
A	CLU	clusterin	3	6.33 × 10^−2^
A	TF	transferrin	3	6.33 × 10^−2^
A	VIM	vimentin	3	6.33 × 10^−2^
A	CRP	C-reactive protein	4	7.67 × 10^−2^
A	TTR	transthyretin	4	7.67 × 10^−2^
A	APOA4	apolipoprotein A4	3	9.10 × 10^−2^
A	APOC3	apolipoprotein C3	3	9.10 × 10^−2^
A	Apoa1	apolipoprotein A1	2	9.86 × 10^−2^
A	B2M	beta-2-microglobulin	2	9.86 × 10^−2^
A	Irs1	insulin receptor substrate 1	2	9.86 × 10^−2^
A	PON1	paraoxonase 1	2	9.86 × 10^−2^
A	PUM3	pumilio RNA binding family member 3	2	9.86 × 10^−2^
A	Slc2a4	solute carrier family 2 member 4	2	9.86 × 10^−2^
A	VDR	vitamin D receptor	2	9.86 × 10^−2^
B	MFGE8	milk fat globule EGF with factor V/VIII domain	25	2.90 × 10^−8^
B	LALBA	lactalbumin alpha	9	7.72 × 10^−4^
B	LYZ	lysozyme	6	2.24 × 10^−2^
B	CSN2	casein beta	5	1.29 × 10^−2^
B	LTF	lactotransferrin	4	2.66 × 10^−2^
B	MfgE8	milk fat globule EGF with factor V/VIII domain	4	8.48 × 10^−2^
C	IGHE	immunoglobulin heavy episolon chain	4	2.57 × 10^−2^
C	NT5C3A	5 prime-nucleotidase, cytosolic IIIA	3	2.77 × 10^−2^
C	LOC112695262		2	7.67 × 10^−2^
D	FGB	fibrinogen beta chain	3	1.52 × 10^−2^
D	LEP	leptin	3	2.46 × 10^−2^
D	FGG	fibrinogen gamma chain	2	5.11 × 10^−2^
D	Ldlr	low density lipoprotein receptor	2	5.11 × 10^−2^
D	NOS1	nitric oxide synthase 1	2	5.11 × 10^−2^
D	Nos1	murine nitric oxide synthase 1	2	5.11 × 10^−2^
D	TXN	thioredoxin	2	5.11 × 10^−2^
D	APOB	apolipoprotein B	2	7.98 × 10^−2^
E	ABCB1	ATP binding cassette subfamily B member 1	3	1.41 × 10^−2^
E	ABCC2	ATP binding cassette subfamily C member 2	3	1.41 × 10^−2^
E	ABCC3	ATP binding cassette subfamily C member 3	3	1.41 × 10^−2^
E	ABCG2	ATP binding cassette subfamily G member 2	2	4.87 × 10^−2^
E	CASP3	caspase 3	2	4.87 × 10^−2^
E	Gusb	glucuronidase beta	4	7.06 × 10^-−3^
E	HSP90AA1	heat shock protein 90 a family class A member 1	2	7.61 × 10^−2^
E	IL10	interleukin 10	2	4.87 × 10^−2^
E	SLC15A1	solute carrier family 15 member 1	3	1.41 × 10^−2^
E	SLCO2B1	solute carrier organic anion transporter 2B1	2	4.87 × 10^−2^
G	AKT1	AKT serine/threonine kinase 1	3	2.95 × 10^−2^
G	MTOR	mechanistic target of rapamycin kinase	3	2.95 × 10^−2^
G	Crtc1	CREB regulated transcription coactivator 1	2	5.79 × 10^−2^
G	SOD1	superoxide dismutase 1	2	5.79 × 10^−2^
G	TNFSF10	TNF superfamily member 10	2	5.79 × 10^−2^

^1^ Cluster is the same as Table 2 and Figure 4. #/cluster is the number of documents in the cluster containing the protein. *p*-value cut-off was < 0.1. Cluster F had no proteins below this cut-off. Upper case proteins designations are human, and first letter capital followed by lower case designate mouse proteins. The full list of proteins in each cluster and enrichment values are in Supplement File B.

**Table 4:** Disease Associations of Proteins in Thematic Clusters ^1^.

Cluster	#Term ID	Term Description	Observed Protein Count	Background Protein Count	False Discovery Rate
A	DOID:0060158	Acquired metabolic disease	42	320	4.98 × 10^−27^
	DOID:0014667	Disease of metabolism	64	997	1.02 × 10^−26^
	DOID:4	Disease	138	5921	4.54 × 10^−20^
	DOID:9120	Amyloidosis	19	70	1.96 × 10^−16^
	DOID:7	Disease of anatomical entity	109	4452	3.59 × 10^−15^
	DOID:4194	Glucose metabolism disease	21	125	8.46 × 10^−15^
	DOID:0050828	Artery disease	20	118	3.34 × 10^−14^
	DOID:178	Vascular disease	24	223	2.44 × 10^−13^
	DOID:9351	Diabetes mellitus	19	118	3.82 × 10^−13^
	DOID:1287	Cardiovascular system disease	31	454	1.21 × 10^−12^
B	DOID:0014667	Disease of metabolism	32	997	7.14 × 10^−11^
	DOID:0050161	Lower respiratory tract disease	16	206	1.24 × 10^−9^
	DOID:1579	Respiratory system disease	17	263	2.40 × 10^−9^
	DOID:0060158	Acquired metabolic disease	18	320	3.47 × 10^−9^
	DOID:4	Disease	73	5921	1.42 × 10^−8^
	DOID:0050828	Artery disease	12	118	2.09 × 10^−8^
	DOID:850	Lung disease	13	172	8.18 × 10^−8^
	DOID:326	Ischemia	7	23	2.91 × 10^−7^
	DOID:77	Gastrointestinal system disease	19	510	3.18 × 10^−7^
	DOID:552	Pneumonia	7	25	3.81 × 10^−7^
D	DOID:0014667	Disease of metabolism	32	997	6.28 × 10^−13^
	DOID:4	Disease	71	5921	9.45 × 10^−12^
	DOID:0050636	Familial visceral amyloidosis	9	21	4.70 × 10^−11^
	DOID:0060158	Acquired metabolic disease	18	320	2.45 × 10^−10^
	DOID:9120	Amyloidosis	11	70	4.55 × 10^−10^
	DOID:0050828	Artery disease	10	118	1.19 × 10^−6^
	DOID:7	Disease of anatomical entity	52	4452	1.31 × 10^−6^
	DOID:178	Vascular disease	12	223	2.55 × 10^−6^
	DOID:1247	Blood coagulation disease	8	76	7.61 × 10^−6^
	DOID:0050161	Lower respiratory tract disease	11	206	1.00 × 10^−5^
E	DOID:178	Vascular disease	14	223	1.01 × 10^−8^
	DOID:4	Disease	58	5921	1.01 × 10^−8^
	DOID:7	Disease of anatomical entity	50	4452	1.01 × 10^−8^
	DOID:326	Ischemia	7	23	5.33 × 10^−8^
	DOID:77	Gastrointestinal system disease	16	510	9.92 × 10^−7^
	DOID:2914	Immune system disease	17	611	1.37 × 10^−6^
	DOID:0014667	Disease of metabolism	21	997	1.47 × 10^−6^
	DOID:0050828	Artery disease	9	118	2.32 × 10^−6^
	DOID:11162	Respiratory failure	5	10	2.32 × 10^−6^
	DOID:552	Pneumonia	6	25	2.32 × 10^−6^
F	DOID:0060158	Acquired metabolic disease	10	320	1.43 × 10^−7^
	DOID:10652	Alzheimers disease	6	35	1.43 × 10^−7^
	DOID:9351	Diabetes mellitus	7	118	7.14 × 10^−7^
	DOID:9352	Type 2 diabetes mellitus	5	29	9.97 × 10^−7^
	DOID:10763	Hypertension	5	36	2.33 × 10^−6^
	DOID:0014667	Disease of metabolism	12	997	5.77 × 10^−6^
	DOID:0050828	Artery disease	6	118	1.19 × 10^−5^
	DOID:4	Disease	24	5921	1.19 × 10^−5^
	DOID:5844	Myocardial infarction	4	20	1.69 × 10^−5^
G	DOID:18	Urinary system disease	16	315	1.36 × 10^−7^
	DOID:4	Disease	65	5921	1.75 × 10^−7^
	DOID:0050686	Organ system cancer	21	677	2.16 × 10^−7^
	DOID:14566	Disease of cellular proliferation	25	1012	2.40 × 10^−7^
	DOID:850	Lung disease	12	172	3.12 × 10^−7^
	DOID:77	Gastrointestinal system disease	18	510	3.23 × 10^−7^
	DOID:9120	Amyloidosis	9	70	3.23 × 10^−7^
	DOID:162	Cancer	23	895	3.46 × 10^−7^
	DOID:0060158	Acquired metabolic disease	14	320	1.61 × 10^−6^
	DOID:0050687	Cell type cancer	15	406	3.51 × 10^−6^

^1^ Simplified output from DAVID Functional Annotation Tool (https://david.ncifcrf.gov/ (accessed on 30 November 2022). Observed protein counts are the number of proteins from a cluster that map to the term ID/term description. Background is the number of terms in that term ID. The number of diseases with FDR ≤ 10^−5^ was 52, 26, 0, 19, 21, 9, and 25 for clusters A through G, respectively.

**Table 5:** Thematic Cluster versus Documents Words of Selected Documents ^1^.

Ref	Top Document Words	Top Thematic Cluster Words	Cluster
[21]	pattern; dietary; dash; ahei; md; metabolomic; fdr; metabolite; index; framingham	protein, liver, diet, mouse, fatty, rat, disease, obesity, plasma, muscle	A
[22]	diet; score; mortality; cvd; association; p0; incident; mediation; all cause; quality		
[23]	pnald; liver; mitochondrial; glycolipid; deps; parenteral; oxidative; differentially; expressed; patient		
[24]	endothelial; ci; diet; mediterranean; cordioprev; lowfat; coronary; patient; difference; dysfunction		
[25]	nonresponders; curve; responder; body; ketone; roc; glycemic; weight; difference; baseline		
[26]	mfgm; bovine; ifs; specie; globule; fraction; milk; attempt; proteome; fat	milk, protein, peptide, human, infant, milk fat globule membrane, colostrum, lactation, bovine, membrane	B
[27]	mfgm; milk; bovine; colostrum; nglycoproteomes; nglycoproteins; mature; lactation; glycosylation; globule		
[28]	lipid; fat; mfgm; milk; globule; catabolic; membrane; specie; enzyme; utilization		
[29]	phosphorylation; site; mfgm; milk; colostrum; mature; protein; phosphoproteomics; phosphoprotein; globule		
[30]	milk; infant; proteome; ptms; human; expanded; endogenous; mother; like; peptide		
[31]	atopic; glycopeptides; oom; milk; nglycoprotein; rochester; lifestyle; york; mother; child		
[32]	wheat; spelt; bread; flour; ncws; heritability; differed; celiac; protein; hypersensitivities; bread	protein, plant, seed, food, allergen, gluten, wheat, soybean, crop, fruit	C
[33]	tfps; editing; trait; food; plant; improvement; omics; sustainable; nutritious; traditional		
[34]	ibsd; intestinal; irritable; bowel; patient; molecule; tissue; syndrome; tmt; ingestion	gut, probiotic, microbiota, protein, intestinal, cell, microbiome, bacterial, human, host	E
[35]	fasting; intermittent; clock; sunset; day; circadian; dawn; consecutive; neuropsychiatric; syndrome	cell, protein, cancer, meat, colorectal, quality, proteomics, fish, study, muscle	G

^1^ Mapping of specific references (first column) and their top document words to the cluster (last column) document terms. Protein acronyms are defined in Table 3. Some abbreviated terms were left unresolved to provide examples of the search results. In some cases, these terms are filtered for subsequent analysis. References for each paper in a cluster can be obtained by clicking on a dot in Figure S4 of Supplementary file SA.

## Data Availability

All data and results are available in Supplement File SA and SB.

## Supplementary Materials

The following supporting information can be downloaded at: https://www.mdpi.com/article/10.3390/nu15020270/s1, Supplement A: (Supp_A_Monteiro_Morine) and Supplement B: (Supp_B_Monteiro_Morine_Disease_Association). Supplement A is an html file that can be opened by any browser. The file is interactive in that tables contain multiple tabs, can be sorted, and searched. Data in figures are linked to PubMed by hoovering over the bars or dot plots. Supplement B is the output of DAVID functional analysis for disease association of proteins in each cluster.
