# Supplementary material for: Identifying and Analyzing Topic Clusters in a Nutri-, Food-, and Diet-Proteomic Corpus Using Machine Reading
Source: Nutrients. 2023 Jan 5;15(2):270. doi: 10.3390/nu15020270 (PMC9863309; doi:10.3390/nu15020270)
Supplement: Supplementary file 1 [file nutrients-15-00270-s001.zip › Supp_A_Monteiro_Morine_.html]

Proteomics and nutrition query


Code 

- Show All Code
- Hide All Code

# Proteomics and nutrition query

#### Vydiant, Inc.

#### 21 September 2022

# 1 General Information

# 2 Queries

The queries were last updated on 17 August 2022.

## 2.1 PubMed abstracts

**Query 1 (protnutr\_mh):** proteomics[MH] AND “Diet,
Food, and Nutrition”[MH] and Human[MH]

**Query 2 (protnutr\_majr):** proteomics[MAJR] AND “Diet,
Food, and Nutrition”[MAJR] and Human[MH]

**Query 3 (protnutr\_abs):** (proteomics[TIAB] OR “DNA
aptamer”[TIAB] OR Somascan[TIAB]) and (“Nutrition”[TIAB] OR
“Nutritional”[TIAB]) AND (Human[MH] OR Human[TIAB] or individuals[TIAB]
or patients[TIAB] or participants[TIAB] or subjects[TIAB])

## 2.2 PubMed Central full text

**Query 1 (protnutr\_mh):** proteomics[MH] AND “Diet,
Food, and Nutrition”[MH] AND Human[MH] AND (open access[filter] OR
author manuscript[filter])

**Query 2 (protnutr\_majr):** proteomics[MH] AND “Diet,
Food, and Nutrition”[MH] AND Human[MH] AND (open access[filter] OR
author manuscript[filter])

**Query 3 (protnutr\_abs):** (proteomics[Abstract] OR
“DNA aptamer”[Abstract] OR Somascan[Abstract]) and
(“Nutrition”[Abstract] OR “Nutritional”[Abstract]) AND (Human[MH] OR
Human[TIAB] or individuals[Abstract] or patients[Abstract] or
participants[Abstract] or subjects[Abstract]) AND (open access[filter]
OR author manuscript[filter])

**NB1:** Tag [MH] refers to regular MeSH tag; [MAJR]
refers to MeSH tag of primary importance to the paper (as designated by
NCBI staff); [TIAB] refers to ‘title/abstract’. The [TIAB] query
captures, in particular, articles that do not have MeSH tags at all.

**NB2:** Subquery
`(open access[filter] OR author manuscript[filter])` is added
to each query, to limit to those articles freely available to commercial
entities.

# 3 Query results

The above queries combined yielded a total of 965 unique abstracts
and 273 unique full-text records.

Figure 1: Venn diagram showing overlap and unique abstracts from
proteomics-nutrition queries. Each Venn section is linked to the
relevant PubMed records (to a maximum of 500).

Figure 2: Total number of publications by year.

Figure 3: SCIMago Journal Rank of publications in the past 20 years.
A pre-filter on Journal Rank of minimum 2 was set for clarity of
visualization.

# 4 Document annotation

The proteomic-nutrition corpus was annotated using various
approaches, depending on the entity type:

1. Disease annotation: we used the DNorm
   annotation tool from NCBI, which provides integrated functionality for
   disease normalization to **MeSH** IDs.
2. Gene/protein annotation: we used the GNormPlus
   annotation tool from NCBI, which provides integrated functionality for
   gene normalization to **NCBI gene** IDs.

## 4.1 Disease

## 4.2 Gene/protein

# 5 Co-mention analysis

An analysis was performed to identify **sentences**
co-mentioning entity pairs of interest.

## 5.1 Gene/disease

## 5.2 Table of comention statements

The table below includes all comention statements used to produce the
network. Each comention relation is summarized with the following
details:

1. origin sentence
2. tagged entities, labeled as V1 and V1 (vertex 1 and 2). Since our
   comentions are non-directed, there is no semantic difference between V1
   and V2
3. V1 and V2 are further described in terms of:

- text\_found: the exact text representing the given entity
- preflabel: the preferred label for the given entity (which serves to
  collapse synonyms for a common entity). It is important to note that it
  is these preflabels (and not text\_found) that correspond to the nodes in
  the network
- type: i.e., O-antigen, disease, species, anatomy

4. NCT\_code: if available, any NCT code identified in the article
   abstract is indicated here

The preflabel search boxes above the columns can be used to
cross-reference specific nodes in the network. **Note:**
the networks are prefiltered to only include co-mention relations with
more than 5 supporting references. This filter is set for plotting
clarity. The table below contains a more comprehensive set of relations,
requiring only 2 supporting references.

# 6 Document clustering

To gain insight on the thematic content of these papers, we performed
document clustering using the tf-idf metric. Briefly, this metric
describes the *importance* of a given word in a given document
within the context of a larger corpus, such that tf-idf is highest when
a word is common within a given document and rare in the rest of the
corpus. Words with high tf-idf in a given document are therefore loosely
analogous to keywords. We then used these numeric vectors to cluster the
documents into thematic groups with K means clustering (K of 15), as
shown in the table and figure below.

**Note:** Each document cluster is illustrated in a
distinct colour in the plot, with the list of most characteristic words
as the cluster label. These same cluster labels are shown in the table
under the field `cluster topwords`. The most characteristic
words for the single document are shown in the field
`document topwords`.

## 6.1 Cluster summary

## 6.2 Publications by year by cluster

Figure 4: Number of records by year per cluster.

## 6.3 t-SNE cluster figure

**Figure description:** The plot below shows all
documents in the proteomics-nutrition corpus, based on t-SNE
dimensionality reduction of tf-idf document vectors. Each dot refers to
a single article, and documents in close proximity have similar word
(and hence thematic) content. The colour of each dot refers to the
cluster number, and the size refers to SCIMago Journal Rank of
publiction journal. The legend shows the top 10 keywords per cluster,
and therefore can be interpreted as a thematic summary. Click on any
individual legend point to hide/show that cluster in the plot; double
click anywhere in the plot to hide/show the legend; click on any node to
link out to PubMed.


Bokeh Plot
